# Supplementary material for: The genome sequence of the Shepherd’s Fritillary, Boloria pales (Denis & Schiffermüller), 1775 (Lepidoptera: Nymphalidae)
Source: Wellcome Open Res. 2026 Aug 25;10:442. Originally published 2025 Aug 15. [Version 2] doi: 10.12688/wellcomeopenres.24642.2 (PMC13562991; doi:10.12688/wellcomeopenres.24642.2)
Supplement: Peer review history

# Reviewer report: Reviewer response for version 2

**DOI:** 10.21956/wellcomeopenres.30320.r166151

**Published:** 2026-08-31

**Authors:** Jurate De Prins

**Affiliations:** 1 Flemish Entomological Society, Vrieselhof, Belgium

I would like to thank the Wellcome Sanger Institute for inviting me to review this manuscript. I have some comments and would be very grateful if you could take them into consideration: 1. I am very happy to see the subtitle 'species taxonomy'; however, the species name, even in the title of the presented manuscript, is incorrect. Denis and Schiffermüller published their work anonymously in 1775, so the correct citation of the species is as follows: Boloria pales (Denis & Schiffermüller, 1775). The authors refer to Tshikolovets (2011). On p. 490 of this book, the authorship of B. pales is presented as [Schiffermüller], 1775, with the anonymity indicated correctly in square brackets. This way of presenting the species name, indicating the anonymity of the authors in square brackets and presenting the species name correctly from a taxonomic perspective, at least in the title, would be a requirement. If square brackets are not acceptable, the species name can be presented as follows: *Boloria pales *(Denis & Schiffermüller, 1775). This presentation would still be supported by the ICZN.

 2. The other issue to be addressed is the poor state of the specimen presented in Fig. 1. I showed the photograph in Fig. 1 to a couple of entomologists from the Work Group Butterflies of the Flemish Entomological Society, and their confirmation that it is *Boloria pales*, was with reservations (probably, maybe, etc.). The correct identification of species is very important, since mistakes in identification will influence far-reaching future conclusions. The obtained whole genomes and sets of nucleic acids will be used for further different studies, evaluations and decisions.

 3. I cannot comment on the technical protocols, but as far as I can see, they are correct. I am happy to know that the chromosome set consists of 30 pseudomolecules. This number was confirmed in Nymphalids by earlier studies in butterfly karyology.

Are sufficient details of methods and materials provided to allow replication by others?

Partly

Is the rationale for creating the dataset(s) clearly described?

Yes

Are the datasets clearly presented in a useable and accessible format?

Partly

Are the protocols appropriate and is the work technically sound?

Yes

Reviewer Expertise:

Taxonomy, Bioinformatics, Karyology

I confirm that I have read this submission and believe that I have an appropriate level of expertise to confirm that it is of an acceptable scientific standard, however I have significant reservations, as outlined above.

# Reviewer report: Reviewer response for version 2

**DOI:** 10.21956/wellcomeopenres.30320.r165672

**Published:** 2026-08-31

**Authors:** Arun Arumugaperumal

**Affiliations:** 1 Department of Biotechnology, Rajalakshmi Engineering College, Chennai, Tamil Nadu, India

The authors have carried out the corrections mentioned. The genome note can be indexed.

Are sufficient details of methods and materials provided to allow replication by others?

Yes

Is the rationale for creating the dataset(s) clearly described?

Yes

Are the datasets clearly presented in a useable and accessible format?

Yes

Are the protocols appropriate and is the work technically sound?

Yes

Reviewer Expertise:

Genomics; Bioinformatics

I confirm that I have read this submission and believe that I have an appropriate level of expertise to confirm that it is of an acceptable scientific standard.

# Reviewer report: Reviewer response for version 1

**DOI:** 10.21956/wellcomeopenres.27156.r129943

**Published:** 2025-09-16

**Authors:** Arun Arumugaperumal

**Affiliations:** 1 Department of Biotechnology, Rajalakshmi Engineering College, Chennai, Tamil Nadu, India

The authors have presented the genome sequence assembly of *Boloria pales*, commonly known as the Shepherd's Fritillary. The specimen used was recorded as a photograph in figure 1. The authors have used high-end sequencing platform and standard protocols to sequence the butterfly genome. The assembly size reported here is 382.99 Mb. The genome sequence was spread across 30 chromosomal molecules including the Z sex chromosome. Since a female specimen was sequenced, W sex chromosome was also expected. In previously sequenced female members of Boloria genus, like *B. dia* [Ref 1], *B. thore* [ Ref 2], *B. aquilonaris* [Ref 3] and *B. titania* [Ref 4], the ZW sex chromosomes were observed. Under the heading 'Assembly quality metrics', the EBP metric was mentioned as '6.C.Q61'. But in Table 4 it was mentioned as '6.7.Q61'. Correction should be made in this aspect.

Are sufficient details of methods and materials provided to allow replication by others?

Yes

Is the rationale for creating the dataset(s) clearly described?

Yes

Are the datasets clearly presented in a useable and accessible format?

Yes

Are the protocols appropriate and is the work technically sound?

Yes

Reviewer Expertise:

Genomics; Bioinformatics

I confirm that I have read this submission and believe that I have an appropriate level of expertise to confirm that it is of an acceptable scientific standard, however I have significant reservations, as outlined above.

## References

[1] : The genome sequence of the Violet Fritillary, Boloria dia (Linnaeus, 1767) (Lepidoptera: Nymphalidae). *Wellcome Open Research* .2025;10: 10.12688/wellcomeopenres.24636.1 10.12688/wellcomeopenres.24636.1 39944230

[2] : The genome sequence of the Thor’s Fritillary, Boloria thore (Hübner, 1803) (Lepidoptera: Nymphalidae). *Wellcome Open Research* .2025;10: 10.12688/wellcomeopenres.24638.1 10.12688/wellcomeopenres.24638.1 41458311 PMC12739373

[3] : The genome sequence of the Cranberry Fritillary, Boloria aquilonaris (Stichel, 1908) (Lepidoptera: Nymphalidae). *Wellcome Open Research* .2025;10: 10.12688/wellcomeopenres.24620.1 10.12688/wellcomeopenres.24620.1 40786598 PMC12334919

[4] : The genome sequence of the Titania’s Fritillary, Boloria titania (Esper, 1793) (Lepidoptera: Nymphalidae). *Wellcome Open Research* .2025;10: 10.12688/wellcomeopenres.24643.1 10.12688/wellcomeopenres.24643.1 41146894 PMC12553976

# Reviewer report: Reviewer response for version 1

**DOI:** 10.21956/wellcomeopenres.27156.r129944

**Published:** 2025-09-10

**Authors:** Murugan Marimuthu

**Affiliations:** 1 Department of Agricultural Entomology, Tamil Nadu Agricultural University, Coimbatore, Tamil Nadu, India

The data note on “The genome sequence of the Shepherd’s Fritillary, *Boloria pales* (Denis & Schiffermüller), 1775 (Lepidoptera: Nymphalidae)” presents a high-quality chromosomal-level genome assembly of the Shepherd’s Fritillary, *Boloria pales*. The work is part of the Tree of Life initiative and follows standardized protocols for genome sequencing, assembly and curation. The dataset is valuable for future comparative genomics, biogeography, and conservation studies in Lepidoptera. The manuscript is thorough and transparent in methodology, but a few areas could be clarified or expanded to strengthen its scientific impact and accessibility.

 Major comments:       

1. The methods section is highly detailed, with extensive technical descriptions (kits, buffer concentrations, software pipelines). While important for reproducibility, the flow of the main text could be improved by summarizing key steps more concisely and shifting technical details to supplementary material.
2. The assembly is briefly compared to *Boloria selene* for sex chromosome identification. A more detailed comparative perspective with other Nymphalidae genomes (assembly sizes, BUSCO scores, synteny patterns) would increase the biological value of this data note.
3. The assembly contains two haplotypes (382.99 Mb and 364.88 Mb). While this demonstrates technical robustness, the biological implications (heterozygosity, population variation, potential use in evolutionary studies) are not discussed. A short explanation would add relevance.
4. Manual corrections (27 breaks, 66 joins) were made during curation. However, it is unclear how these decisions were validated (Hi-C contact map evidence, synteny with related species). A brief justification would enhance transparency and confidence in the assembly.
5. How do the authors interpret the finding of a **Z0 karyotype** in this female specimen? Could this represent a genuine biological feature, an assembly artifact, or incomplete phasing?
6. The mitochondrial genome was assembled—could the authors provide **annotation details** (genes, control region, synteny with related butterflies)
7. Whether any **contaminant sequences were removed** during ASCC decontamination? If yes, what types of contaminants were found?
8. The “Genome sequence report” and “Assembly quality assessment” overlap in some results. They can be merged or cross-referenced to avoid redundancy.

Minor comments:

1. GenomeScope estimated the haploid genome size at 394.75 Mb, while the final assembly was 382.99 Mb. A brief note on this discrepancy (collapsed repeats, heterozygosity, missing sequence) would be helpful.
2. Some figures (BlobToolKit plots, BUSCO snail plot) are not fully explained in the legends. Consider adding short descriptions to help non-specialist readers interpret them.
3. Ensure consistent italicization of species names ( *Boloria pales*). Some numbers have formatting artifacts (“1 500 m” instead of “1,500 m”). Several links are repeated multiple times in succession and could be consolidated.
4. The k-mer completeness values (67.56% haplotype 1, 64.75% haplotype 2, 98.88% combined) may not be familiar to all readers. A one-sentence interpretation in simple language would be useful.
5. Figures 2–6 are informative, but legends could provide clearer biological interpretations (e.g., “Figure 3 shows chromosomal painting, highlighting conserved linkage groups relative to ancestral Lepidopteran karyotypes”).
6. Ensure consistent formatting of numbers, units (m, kb, Mb, Gb), and percentages.

Are sufficient details of methods and materials provided to allow replication by others?

Yes

Is the rationale for creating the dataset(s) clearly described?

Yes

Are the datasets clearly presented in a useable and accessible format?

No

Are the protocols appropriate and is the work technically sound?

Yes

Reviewer Expertise:

Insect omics and their application in pest management

I confirm that I have read this submission and believe that I have an appropriate level of expertise to confirm that it is of an acceptable scientific standard, however I have significant reservations, as outlined above.
